# A hybrid digital parenting programme to prevent abuse of adolescents in Tanzania: study protocol for a pragmatic cluster-randomised controlled trial

**DOI:** 10.1186/s13063-023-07893-x

**Published:** 2024-02-13

**Authors:** Lauren Baerecke, Abigail Ornellas, Joyce Wamoyi, Mwita Wambura, Jonathan Klapwijk, Angelique N. Chetty, Ashlin Simpson, Roselinde Janowski, Kristen de Graaf, David Stern, Lily Clements, Esmee te Winkel, Laetitia Christine, Gervas Mbosoli, Kija Nyalali, Onduru Gervas Onduru, Anna Booij, Sussie N. Mjwara, Sibongile Tsoanyane, Gerry Mshana, Mbutolwe Esther Mwakitalu, G. J. Melendez-Torres, Francisco Calderon, Isang Awah, Ohad Green, Inge Vallance, Oluwaseyi Somefun, Frances Gardner, Lorraine Sherr, Mackenzie Martin, Jamie M. Lachman, Lucie D. Cluver

**Affiliations:** 1 Safety and Violence Initiative, Centre for Social Science Research, University of Cape Town, Cape Town, South Africa; 2 National Institute for Medical Research, Mwanza Research Centre, Mwanza, Tanzania; 3 Department of Social Policy and Intervention, University of Oxford, Oxford, UK; 4 Innovations in Development, Education and the Mathematical Sciences (IDEMS) International, Reading, UK; 5 Innovations in Development, Education and the Mathematical Sciences (INNODEMS), Kakamega, Kenya; 6 Clowns Without Borders South Africa, Durban, South Africa; 7 Faculty of Health and Life Sciences, University of Exeter, Exeter, UK; 8 The Haruv Institute, Jerusalem, Israel; 9 School of Public Health, University of the Western Cape, Cape Town, South Africa; 10 Institute for Global Health, University College London, London, UK; 11 Parenting for Lifelong Health, Oxford, UK; 12 Department of Psychiatry and Mental Health, University of Cape Town, Cape Town, South Africa

**Keywords:** Child abuse, Parenting, Adolescents, Digital, Low- and middle-income countries, Violence against children

## Abstract

**Background:**

Evidence-based parenting programmes have strong evidence in preventing and mitigating violence, but in-person programmes are challenging to deliver at scale. ParentApp is an open-source, offline-first app-based adaptation of the Parenting for Lifelong Health for Parents and Teens programme to promote playful and positive parenting, reduce risks for sexual violence victimisation, and prevent violence against adolescents. This study aims to evaluate the effectiveness and cost-effectiveness of ParentApp compared to an attention-control group.

**Methods:**

This study is a two-arm pragmatic cluster-randomised controlled trial to test whether ParentApp reduces adolescent physical abuse, emotional abuse, and sexual violence risks and victimisation at 1 month and 12 months post-intervention. Caregivers of adolescents aged 10–17 years and their adolescent children (*N* = 2400 caregiver-adolescent dyads) will be recruited in urban and peri-urban communities in the Mwanza region of Tanzania. A total of 80 study clusters will be stratified and randomised (1:1) to the intervention group, who will receive ParentApp with support through a WhatsApp group, or to an attention-control group, who will receive a water, sanitation, and hygiene app. Quantitative data will be collected through outcomes questionnaires with caregivers and adolescents, administered at baseline, 4 months post-baseline, and 16 months post-baseline, as well as through routine implementation data and ParentApp engagement data. Qualitative data will be collected through individual interviews and focus groups with caregivers, adolescents, and implementing partner staff.

**Discussion:**

App-based interventions have the potential to expand access to evidence-based parenting support, but currently lack rigorous evidence in low- and middle-income countries. This is the first known randomised control trial of a hybrid digital parenting programme to prevent the abuse of adolescents in low- and middle-income settings.

**Trial registration:**

The trial was registered on the Open Science Framework on 14 March 2023, registration: OSF.IO/T9FXZ.

## Administrative information

Note: the numbers in curly brackets in this protocol refer to SPIRIT checklist item numbers. The order of the items has been modified to group similar items (see http://www.equator-network.org/reporting-guidelines/spirit-2013-statement-defining-standard-protocol-items-for-clinical-trials/).

**Table:** 

Title {1}	A hybrid digital parenting programme to prevent abuse of adolescents in Tanzania: study protocol for a pragmatic cluster randomised controlled trial
Trial registration {2a and 2b}	The trial was registered on the Open Science Framework on 14 March 2023, registration: OSF.IO/T9FXZ (10.17605/OSF.IO/T9FXZ)
Protocol version {3}	2
Funding {4}	This study is funded by the UK Research and Innovation Global Challenges Research Fund Accelerating Achievement for Africa's Adolescents (Accelerate) Hub (ES/S008101/1), the LEGO Foundation, Oak Foundation (OFIL-21–212), the European Research Council, with the UK Research and Innovation Engineering and Physical Sciences Research Council PROTECT (EP/X039307/1), and a private family trust which wishes to remain anonymous. This study is part of the Global Parenting Initiative, which is funded by the LEGO Foundation, Oak Foundation, the World Childhood Foundation, and The Human Safety Net. We thank Vodacom Tanzania for support with low-cost smartphones and mobile data
Author details {5a}	^1^ Centre for Social Science Research, University of Cape Town, Cape Town, South Africa ^2^ National Institute for Medical Research, Mwanza Research Centre, Tanzania ^3^ Department of Social Policy and Intervention, University of Oxford, United Kingdom ^4^ Innovations in Development, Education and the Mathematical Sciences (IDEMS) International, United Kingdom ^5^ Innovations in Development, Education and the Mathematical Sciences (INNODEMS), Kenya ^6^ Clowns Without Borders South Africa, South Africa ^7^ Faculty of Health and Life Sciences, University of Exeter, United Kingdom ^8^ The Haruv Institute, Israel ^9^ School of Public Health, University of the Western Cape, South Africa ^10^ Institute for Global Health, University College London, United Kingdom ^11^ Parenting for Lifelong Health, United Kingdom ^12^ Department of Psychiatry and Mental Health, University of Cape Town, South Africa
Name and contact information for the trial sponsor {5b}	University of Oxford Department of Social Policy and Intervention Barnett House, 32–37 Wellington Square, Oxford OX1 2ER Telephone: + 44 1,865,270,325 National Institute for Medical Research (NIMR) P.O. Box 1462, Mwanza, Tanzania Telephone: + 255 282,500,399 University of Cape Town (UCT) Centre for Social Science Research Robert Leslie Social Science Building, Rondebosch 7701 Telephone: + 27 216,504,656
Role of sponsor {5c}	The study was co-designed by University of Oxford, NIMR and UCT as equal sponsors Funders played no role in the study design and will not be involved in its execution, analyses, interpretation of data, or dissemination of results

## Introduction

### Background and rationale {6a}

Ending violence against children has been identified as a strategic objective for global sustainable development [1]. The global prevalence of violence against children is estimated at one billion children a year; this represents over half of the global child population ages 2–17 years [2]. Rates of violence against children are particularly high in low- and middle-income countries. The African continent has one of the highest global rates of physical and verbal abuse of children and adolescents [3], and the prevalence of sexual violence during childhood is between 22 and 38% across sub-Saharan African countries [4]. Violence against children is associated with multiple negative outcomes across the life course, including health risks, substance abuse, mental health distress, and low academic performance [5]. Long-term, violence against children has been linked to increased mortality, unemployment, crime, and intergenerational violence [6]. The societal impact of violence against children includes increased burden on health and welfare services and reduced economic productivity [7]. Estimates of global violence against children costs are as high as USD 7 trillion [8].

The 2023 WHO Guidelines on Parenting Interventions identifies that evidence-based parenting programmes are effective at reducing and preventing violence against children [9]. Parenting interventions work by strengthening protective factors (e.g. alternatives to physical punishment) and reducing risk factors (e.g. disruptive child behaviour and parental stress) [10–12]. Systematic reviews have identified over 400 randomised trials, with 145 in low- and middle-income countries, showing successful outcomes of in-person parenting programmes in lowering the risk of violence against children [13–16]. Cost-effectiveness analyses show parenting interventions to be an effective and cost-effective approach for reducing violence against children, particularly when compared to other intervention types [17, 18].

Parenting for Lifelong Health (PLH) is a collaborative initiative established by academics across the Global South and North, with the World Health Organisation and UNICEF, and in collaboration with USAID-PEPFAR and others. Since 2012, PLH has developed, tested, and supported the delivery of a suite of open-source, evidence-based parenting programmes for infants, children, and adolescents. Programmes are designed with families in low- and middle-income countries and optimised to reduce costs of delivery. Fifteen randomised trials of PLH programmes in low- and middle-income countries have demonstrated significant effects on reduced violence against children and women, financial insecurity, and family safety planning, while improving positive parent–child relationships, mental health, and parenting behaviours [19–21]. Even though PLH programmes have focused on improving parent–child relationships, evaluations of PLH programmes have shown reduced co-occurrence of violence against children and intimate partner violence (IPV). An evaluation of a PLH adaptation for caregivers of young children in the Philippines found a significant reduction in caregiver-reported IPV, hypothesised to relate to improved confidence in women’s relationships with their partners and other adults in the household and to the mindfulness activities that are part of PLH programmes [19]. Parenting programmes, particularly those that engage men and support engagement between fathers and their children, have been identified as a promising strategy for addressing norms that contribute to both violence against children and women, and preventing IPV [22–24].

PLH for Parents and Teens (PLH Teens) is part of the PLH suite of programmes targeting parents and adolescents ages 10–17 years. Originally developed in South Africa [25], it aims to reduce adolescent exposure to violence in the home and community by improving positive parenting, parent–child communication, mental health, and by reducing familial conflict, harsh discipline, parenting stress, and reducing risks for sexual violence victimisation. PLH Teens is amongst the few low-cost parenting interventions for families with adolescents that has been rigorously tested and disseminated in low- and middle-income countries [18]. This includes testing in rural villages and peri-urban townships in South Africa through a cluster-randomised controlled trial (RCT) which demonstrated significant impacts, improving positive parenting practices, reducing child abuse, and improving family finances [20].

There are, however, challenges in delivering in-person parenting programmes to the scale needed, particularly in terms of human resource and economic cost [26]. Barriers to implementing in-person programmes were further exacerbated with the onset of the COVID-19 pandemic [27], as national lockdowns and health emergencies made in-person programmes difficult to deliver. Simultaneously, the pandemic has caused unprecedented stress for families globally, through loss of family members, mental distress, isolation, school closure and an economic crisis still felt today [28, 29]. As a result, physical and emotional violence against children, as well as sexual exploitation, has risen [30].

Digital or hybrid digital interventions, which combine remote or in-person human support with digital delivery, may be a valuable delivery approach to increase scale-up of parenting programmes at national levels. Evidence generation is still in the very early stages. A systematic review and meta-analysis identified 15 randomised trials of digital parenting programmes [31]. Online interventions were found to be effective in reducing child behaviour problems and improving parenting behaviours, with comparable effectiveness on child behaviour to traditionally delivered parenting programmes. However, these were mostly small studies in the Global North with young children. The expansion of programme delivery via digital approaches is supported by regional trends showing exponential smartphone access and reduced data costs over the next decade in sub-Saharan Africa. Smartphone ownership is estimated to rise from 37% in 2017 to around 67% in 2025 [32].

ParentApp is a mobile application specifically designed to deliver a remote version of the in-person PLH programme in settings where there is no internet, or where internet connection is sporadic, and on low-cost local smartphones. Once downloaded, the app can deliver the programme without a connection to the internet. From 2019 to 2022, ParentApp was co-developed and optimised for caregivers of adolescents aged 10–17 years in seven stages:

1. Multi-phased adaptation and co-development with researchers, programme specialists, and technical experts, as well as PLH Teens participants and facilitators from a rural community in South Africa;
2. Participatory engagement with 518 participants (caregivers, adolescents and implementing staff) in 14 countries across Africa and Asia who received COVID-19 parenting resources [33];
3. Mixed methods user-testing study with 24 participants (18 caregivers and 6 adolescents) from nine African countries [34];
4. Feasibility pilot of an early beta version of ParentApp delivered as a self-guided programme with 107 caregivers and nine implementing staff in South Africa;
5. Quantitative analysis of a pre-post pilot-test with 204 participants (103 caregivers and 101 adolescents) in rural and urban Tanzania;
6. Cluster-randomised factorial trial in urban and peri-urban Tanzania with 614 caregivers, using a 2 × 2 × 2 multifactorial experimental design to examine the effectiveness and cost-effectiveness of three intervention components aimed at enhancing engagement: Support (self-guided/moderated WhatsApp groups), app design (sequential workshops/non-sequential modules), and a brief digital orientation session (on/off) [35]; and
7. Two qualitative studies, one with 39 participants (21 caregivers and 18 adolescents), drawn from the pilot-test; and one with 38 caregivers drawn from the cluster-randomised factorial trial.

Content and delivery for the Tanzanian version of ParentApp were jointly developed and adapted through the steps outlined above by the Universities of Oxford and Cape Town, IDEMS International, Tanzania’s National Institute for Medical Research (NIMR), PLH, and Clowns Without Borders South Africa (CWBSA). Implementation was supported by Tanzania-based NGO Investing in Children and Strengthening their Societies (ICS).

#### Policy context and partnership

A national survey on violence against children in Tanzania showed nearly three quarters of children (13–17 years) experienced physical violence by a relative, partner or authority figure in the past year [36]. Caregivers and other adult relatives are the most reported perpetrators of physical and emotional violence against children. An estimated 27% of girls and 12% of boys in Tanzania have experienced sexual violence before the age of 18 years [37]. As a Pathfinder Country, Tanzania has prioritised ending violence against children and has identified parenting support as a key intervention strategy. The National Plan of Action (NPA-VACW, 2017/2022) includes parenting as one of its eight thematic areas in preventing and responding to violence against children [38].

The in-person PLH Teens programme (locally known in Tanzania as *Furaha Teens*) was successfully delivered to 75,061 Tanzanian caregivers and children with promising positive impact in a large-scale pre-post evaluation, including reduced child maltreatment, improved family relationships, promoted child development, and reduced IPV perpetration and experience [39]. The Tanzanian-adapted version of PLH Teens also demonstrated high cultural acceptability and programme fidelity when delivered at scale. However, cost and human resources limit the capacity for in-person parenting programmes to be scaled up to all families in Tanzania. During the early piloting of ParentApp in Tanzania in 2022, the national government announced their intentions to deliver ParentApp as a country-wide programme to support families within their National Framework.

As part of the rigorous evaluation of ParentApp, the study team will work with the Government of Tanzania to develop an evidence-based approach to parenting support at scale. The National Institute for Medical Research will support the government and other stakeholders—including the UNICEF Country Office, WHO country office, and USAID-PEPFAR—to develop a strategic action plan to adapt, deliver, and sustain evidence-based parenting solutions. Policy engagement focuses on scaling evidence-based, open-source parenting support through various approaches including in-person, remote, digital, hybrid, and multimedia modalities from multiple programmes. Processes will include formation of a Parenting Scale Up Consortium, stakeholder mapping and analysis, formation of advisory groups, and in-person and virtual convenings with stakeholders [40].

This policy and evidence-building takes place within a global policy context, as part of the Global Initiative to Support Parents. This is an alliance of WHO, UNICEF, the Global Partnership to End Violence Against Children, the Early Childhood Development Action Networks and PLH, with the shared goal of ensuring universal access to evidence-based parenting support.

### Objectives {7}

The study aims to test the effectiveness and cost-effectiveness of an offline-first, open-source parenting application for caregivers of adolescents aged 10–17, delivered with WhatsApp groups to support engagement, against an attention-control group, in low-income urban and peri-urban settings in Tanzania. The study has the following objectives: (1) to test the effectiveness and cost-effectiveness of ParentApp on reducing adolescent maltreatment and sexual violence risk and victimisation; (2) to test delivery of ParentApp at scale through local implementing partners; (3) to explore pathways of impact through mediation and moderation models and qualitative investigation; and (4) to co-develop a national strategy with the Government of Tanzania to scale evidence-based parenting support.

Based on these objectives, the following hypotheses were developed:

- Hypothesis 1: ParentApp will reduce rates of adolescent physical and emotional abuse, and sexual violence risk and victimisation, in comparison to an attention-control group, at 1 month and 12 months post-intervention.
- Hypothesis 2: ParentApp will have a significant effect on outcomes related to the risk of adolescent physical and emotional abuse, including improving positive parenting; reducing dysfunctional parenting; improving child monitoring and supervision; reducing parental mental health distress; reducing child neglect; reducing child behaviour problems; reducing endorsement of physical punishment; improving parent-adolescent relationships; improving caregiver support for school; improving gender equitable behaviours; improving attitudes towards gender roles and IPV; reducing parental stress; improving parent–child communication; improving financial self-efficacy and family financial management; improving social support; reducing sexual risk behaviours; and reducing intimate partner violence experiences, in comparison to an attention-control group, at 1 month and 12 months post-intervention.

Potential pathways that have been shown to mediate change in primary outcomes in studies of parenting interventions aimed at reducing violence against children will be examined. Potential moderators of programme effects will also be explored. Given that no known randomised trials have evaluated hybrid digital parenting interventions in low- and middle-income countries, we do not advance additional mediator or moderator hypotheses that pertain to caregiver, adolescent and family characteristics. All mediator and moderator models will be exploratory.

### Trial design {8}

This is a two-arm, pragmatic, cluster-randomised controlled trial with assessments at baseline, 1 month post-intervention and 12 months post-intervention. Randomisation will be performed at the cluster level with a 1:1 allocation ratio.

A total of 80 urban and peri-urban clusters in Mwanza, Tanzania, containing on average 30 caregiver-adolescent dyads each, will be randomised to two parallel arms. Caregivers in intervention sites will receive a basic smartphone, which will be loaded with the Kiswahili version of ParentApp and will be invited to join a WhatsApp group to discuss and share solutions as they use the app. Caregivers in control sites will also receive a basic smartphone, which will be loaded with a Kiswahili app giving water, sanitation, and hygiene (WASH) information and WhatsApp.

## Methods: participants, interventions, and outcomes

### Study setting {9}

Eligible communities for the trial are urban and peri-urban sub wards in Tanzania’s Mwanza City, with at least 30 households that are eligible to participate in the national Tanzania Social Action Fund (TASAF) programme. Eligibility was selected as a nationally recognised marker of poverty and vulnerability, although only a sub-set of eligible families receive the programme. We will use a simplified version of the eligibility criteria [41]. We will also target households that do not own smartphones.

### Eligibility criteria {10}

Three types of participants will be recruited for the trial—caregivers, adolescents, and implementing partner staff. Caregivers and adolescents will be recruited from eligible clusters, which include sub wards in the Mwanza region. Sub wards are defined as the lowest administrative structure at the community level in urban settings in Tanzania.

Inclusion criteria for participating caregivers within the selected clusters:

- Age 18 years or older,
- Primary caregiver currently caring for an adolescent between the ages of 10 and 17 years,
- Staying in the same household as the adolescent at least four nights a week in the past month,
- Basic literacy, which will be determined during screening using the following question: “How do you find reading?”. Participants who respond that they “can read with a little difficulty” or “can read easily” will be included whereas those who respond that they “can’t read at all” or “can read but with lots of difficulty” will be excluded.
- In agreement to participate in either the ParentApp (intervention) or WASH App (control) programme, and
- Has provided written and/or oral informed consent to participate in the full study.

Inclusion criteria for participating adolescents:

- Age 10–17 years,
- The dependent of a parent or caregiver who meets the above inclusion criteria and participates in the trial,
- Has provided written and/or oral assent to participate in the full study, and
- Primary caregiver has provided written and/or oral consent for the adolescent to participate in the full study.

If there are multiple adolescents in the household, the caregiver is asked to select the adolescent whose ‘behaviour is giving them the most problems.’ Selection of the index teen is not based on gender and there are no efforts to balance the number of male and female adolescents in the trial.

Implementing partner staff will be eligible to participate in the study if they are:

- Aged 18 or older,
- Previous participants of the ParentApp facilitator training workshop, and
- Able to provide written and/or oral informed consent to participate in the study.

Any participant exhibiting severe mental health problems or acute mental disabilities that mean they are unable to give informed consent will be excluded from the study for ethical reasons.

### Who will take informed consent? {26a}

Informed consent procedures will be conducted by a trained field research team at each data collection point to allow participants to decline to participate at any stage. Consent procedures will include clear descriptions of the study objectives, participant involvement, engagement with and protection of participant data, and the participant’s right to withdraw consent at any point in the study. For adolescents to participate, primary caregivers must provide their consent and adolescents must provide their assent. Information and consent processes will be age appropriate, conducted in the language of the participant’s choice, and will be read aloud in cases of low literacy.

### Additional consent provisions for collection and use of participant data and biological specimens {26b}

The informed consent procedure for engagement data collected through the control and intervention apps will be included in the study consent form. In addition, before using the apps, participants will be required to accept the Terms and Conditions and Privacy Policy, which contain reminders of how personal information and app engagement data will be collected, used, and shared.

### Interventions

#### Explanation for the choice of comparators {6b}

An alternative content comparator was selected. Participants in the active control group will receive an app that contains information on WASH, with a similar number of modules and similar app design to ParentApp. Provision of smartphones and data will facilitate similar levels of digital access for participants in the intervention and control groups, as well as app use. The control group will be supported to download WhatsApp on their phones although they will not participate in a WhatsApp support group.

#### Intervention description {11a}

ParentApp is an open-source mobile application that delivers content through 12 interactive core modules covering the topics listed in Table 1. The content focuses on building trusting relationships between parents and adolescents, managing difficult behaviour, non-violent approaches to discipline, problem solving, and family budgeting. The app provides home practice activities for caregivers to apply key parenting skills from each module with their adolescents. Caregivers can log positive parenting behaviours through a habit-tracking tool called ‘ParentPoints’ and have a library with access to local support resources, information, and technical support.

**Table 1:** ParentApp modular content

Module	Topic
1	Parental self-care and stress reduction
2	One-on-one time
3	Praise and positive reinforcement
4	Positive instructions
5	Managing stress
6	Family budgeting
7	Rules
8	Consequences and accepting responsibility
9	Problem solving
10	Teen safety
11	Dealing with crisis
12	Celebration and next steps

In response to piloting and requests from families, adaptations were made to the programme content to include (1) content to support caregiver mental health through mindfulness techniques, (2) content for families experiencing bereavement, (3) content to support playful interactions to promote parent-adolescent relationship building, and (4) enhanced evidence-based content to prevent and respond to risks of sexual and online violence including No Means No Worldwide programme content [42]. There is limited evidence of the impact of parenting programmes on sexual violence against children, especially in LMICs. However, parenting and positive parent–child relationships are important protective factors in reducing risks of child sexual violence through improving parental supervision and monitoring and promoting child self-efficacy and wellbeing [43, 44]. ParentApp includes content on online and offline safety planning and parent–child communication about risk and disclosure, which are promising pathways to protect against sexual violence experience and risk [45]. We have further included adapted content from No Means No Worldwide, an empowerment self-defence programme that has been shown to reduce sexual violence victimisation amongst adolescents girls [42].

As the piloting and cluster-randomised factorial trial of ParentApp identified that moderated WhatsApp groups contribute to improvements in engagement during app-based delivery, caregivers will join WhatsApp support groups with other caregivers from their study cluster. There will be one WhatsApp group per cluster and each group will be moderated by one lead facilitator and one co-facilitator. Locally recruited professionals and non-professionals (e.g. peer parents, teachers) will serve as facilitators. Facilitators will be trained by Clowns Without Borders South Africa, an organisation which has successfully led training of PLH programmes internationally and in Tanzania. There is a facilitator manual which guides facilitators on how to deliver WhatsApp-based support and provides standardised messages to be sent for each module. Facilitators will monitor discussions, share tips and reminders, and lead weekly 1-h live chat sessions. Live chat sessions focus primarily on sharing caregiver experiences with home-based practices and activities. Facilitators will receive supervision by trained coaches during the intervention delivery period.

ParentApp content is flexible, and caregivers can move through the twelve modules at their own pace and in their own time. However, WhatsApp support focuses on one primary module each week, over a 12-week period, consecutively from module 1 through 12. In week 13 and 14, facilitators will send encouraging messages to caregivers to complete the final modules if they have not done so. Participants will continue to have access to ParentApp’s full content for the duration of the study.

#### Criteria for discontinuing or modifying allocated interventions {11b}

Caregivers and adolescents may withdraw from the study and/or interventions at any time without any consequences. All data up to the point of withdrawal will be retained unless requested otherwise. If we determine that participants or their families have experienced significant harm (i.e. abuse, suicidality, intimate partner violence, or other potential psychological or physical injuries) as a result of participation in the study or interventions, we will cease activities until these issues can be addressed adequately.

#### Strategies to improve adherence to interventions {11c}

Caregivers in the intervention and control groups will receive a low-cost locally sourced smartphone for use during the trial. While smartphone access in Tanzania is currently at 38%, the telecommunications sector is experiencing rapid growth, and the government plans to increase smartphone-based internet access to 80% of the population by 2025 [46]. Following pragmatic trial principles [47], we therefore aimed for a sample that reflects smartphone access levels at the trial end date of 2025, rather than at the start date of 2023. Caregivers will be able to keep the phone after the end of the study.

ParentApp stores digital engagement data, which will be shared automatically when the device is connected to the internet and monitored during the trial. To collect this data, as well as participate in WhatsApp activities where relevant, caregivers will be provided with monthly data bundles for the duration of the study to enable app usage and data syncing, up to 1 GB total per month (around $0.70).

#### Relevant concomitant care permitted or prohibited during the trial {11d}

Participants are not restricted from receiving other care during the trial period.

#### Provisions for post-trial care {30}

The risk of participants experiencing any adverse events during this trial because of the interventions is very low. However, we will employ safeguarding procedures to mitigate risks of adverse effects. Where any participant discloses information which shows them or others to be at risk of significant harm, the research team will make direct referrals to relevant service providers. The referral process will be triggered through participant survey responses that indicate potential harmful experiences and/or support needs (e.g. violence, severe mental ill-health, neglect). Direct referrals to in-person social services will also be available through the implementing partner, should these be requested by participants. ParentApp includes national self-referral and emergency contacts which are affordable and remote-friendly, should families require further support during or after the trial. These resources were identified by the research and implementing teams following a thorough mapping of online and affordable support systems in Tanzania.

### Outcomes {12}

#### Adolescent and caregiver outcomes

Collection of data on adolescent and caregiver outcomes will take place at three time points: baseline, 1 month post-intervention (4 months post-baseline), and 12 months post-intervention (16 months post-baseline). Outcome measurements will be reported independently by caregivers and adolescents, unless specified otherwise. Standardised measurements and standard timeframe of events or practices over the “past 4 weeks” will be used to strengthen study validity and identify potential changes in behaviour due to the intervention. The outcomes will be informed by behaviour change theoretical components and the veracity of these will be assessed empirically. All measures are translated into Kiswahili and back translated to check accuracy. Some measures have adaptations made for comprehensibility and contextualisation, after feedback from their use in the pre-post pilot for this trial.

##### Primary outcomes

*Child maltreatment (physical and emotional abuse)* will be assessed using the relevant subscales of the Caregiver and Child Versions of the International Society for the Prevention of Child Abuse and Neglect Screening Tool for Trials (ICAST-Trial) [48]. *Sexual violence risk and victimisation* (adolescent report only) will combine measures of contact and non-contact sexual abuse, and exposure to high-risk situations for abuse, and be evaluated using a combination of items from different tools: the ICAST-Trial [48], measures used in the CDC Violence Against Children and Youth Surveys [49], and a locally derived risk scale developed in consultation with families and practitioners.

##### Secondary outcomes

- *Poor parental supervision*, *inconsistent discipline*, *positive parenting* and *positive involved parenting* will be assessed using the relevant subscales from the Alabama Parenting Questionnaire [50].
- *Neglect* will be measured using an adapted version of the neglect subscale from the ICAST-Trial Caregiver and Child Versions [48].
- Measures of caregiver and adolescent *attitudes to physical punishment* using an item from the UNICEF Multiple Indicator Cluster Surveys (MICS) 5 Child Discipline module [51].
- *Family-level planning to reduce risks of sexual violence* victimisation will be assessed using an item developed in South Africa for a randomised trial of the in-person PLH Teens programme [20].
- *Intimate partner violence (IPV) experience, witnessing* (adolescent report only) and* perpetration* (caregiver report only) will be measured using items adapted from the WHO’s Violence Against Women Instrument (VAWI) used in the WHO Multi-Country Study on Domestic Violence [52], and items from the ICAST-Trial Child Version [48].
- *Gender equitable behaviours* (caregiver report only) will be measured using four items adapted from questionnaires used in previous violence prevention studies: Three items are adapted from a questionnaire that was developed by researchers at the London School of Hygiene and Tropical Medicine (LSHTM) and used in an RCT of a violence prevention intervention in Tanzania [53]. The fourth item is adapted from a questionnaire developed by researchers of an RCT of a gender-transformative violence prevention intervention in Rwanda [54].
- Caregiver and adolescent *attitudes towards gender roles and IPV* will be measured by self-report using items adapted from the ‘Attitudes towards gender roles’ section of the WHO Multi-Country Study on Domestic Violence [52].
- *Caregiver support for school* is measured through five items assessing parent involvement and support of education [55].
- *Parent communication* is measured using items adapted from the Parent–Child Communication Scale used in the Fast Track Intervention Study [56].
- *Economic hardship* is measured using items on monthly shortfalls of basic necessities, such as clothes, soap, and school equipment (reported independently by both caregivers and adolescents).
- *Financial self-effi*cacy and *family financial management* are measured using items on borrowing (from loan sharks and others), saving and budgeting.
- *Parenting stress* (caregiver report only) will be assessed using the Parental Stress Scale [57].
- Caregiver and adolescent *mental health distress* will be evaluated using the Patient Health Questionnaire 4 (PHQ-4), a brief four-item screening for symptoms of anxiety and depression [58].
- *Social support* for caregivers (reported by caregivers) and adolescents (reported by adolescents) will be measured using the Medical Outcomes Study Social Support Survey [59].
- *Sexual risk behaviour* (adolescent report only) measures are adapted from the CDC Violence Against Children and Youth Surveys (VACS) and the South African Demographic and Health Survey [60]. One item on age-disparate sex was included.
- Components will be interrogated in alignment with theoretical models of behaviour change.

##### Other pre-specified and exploratory outcomes

*Use of internet and exposure to online violence risk* will be evaluated using items adapted from the Global Partnership to End Violence Against Children’s Disrupting Harm project [61]. *Digital literacy*, measured by questions developed by the research team, will assess technical, social, critical, and creative digital skills. *Adolescent externalising and internalising behaviour* are measured using the Child and Adolescent Behaviour Inventory (CABI) [62] and reported by caregivers only. *Substance use* is reported by caregivers and adolescents using items adapted from the WHO Alcohol Use Disorders Identification Test [53] and the WHO Global School-based Health Survey [54].

The concept of *engaged responsive parenting* will be used as a uniting concept covering the cross-cultural indicators of playful learning [63], characteristics of learning through play [64, 65], and positive parenting. Engaged responsive parenting will be measured by combining items from the APQ positive parenting and involvement subscales, the parent–child communication scale (caregiver only) and parent support for school. Caregiver’s engagement with playful activities in the app (e.g. number of playful activities completed) will be measured through app engagement data.

#### Socio-demographic measures

Socio-demographic variables measured at baseline include age, gender, assessment of disability (also assessed at 1 month follow-up), assessment of basic literacy, household structure, adolescent’s relationship to the caregiver, household employment, school enrolment, relationship status and orphanhood. Demographic factors to be measured include general questions as well as items from the Multiple Indicator Cluster Surveys (MICS) [51]. We will assess access to social protection (e.g. government cash transfers) to explore whether combined social protection and parenting support is associated with improved parent and child outcomes.

#### Cost-effectiveness outcomes

Cost-effectiveness outcomes will include facilitator and participant app engagement, measuring time spent and data costs incurred, as well as delivery costs (e.g. provision of devices, facilitator training and support, app maintenance, programme onboarding) and intervention costs (e.g. WhatsApp group support). Cost data will be collected through weekly Open Data Kit (ODK)-based surveys with facilitators. Facilitators will record time and data usage of prescribed intervention efforts, as well as basic participant engagement statistics. A visual analogue scale from 0 to 100, administered at baseline and follow-up with caregivers and adolescents, will be used to approximate quality-adjusted-life-years (QALY) to support long-term cost-effectiveness measures. Modelling maltreatment outcomes over time and the associated QALYs linked to these will support long-term cost-effectiveness analyses. We will adopt a multi-outcome discounted cost-effective analysis methodology, in order to better understand the cost and savings of a single intervention (ParentApp) across multiple sectors and violence against children pathways [66, 67].

#### Implementation outcomes

Engagement data, such as the amount, frequency, and duration of usage, is collected automatically through the intervention and control apps and used to examine programme fidelity, exposure, and adherence. It includes measures of engagement with the app (e.g. number of times the app is opened, time spent on the app), engagement with the content and skills (e.g. number of modules completed, number of activities completed, positive behaviours logged), and other engagement measures (e.g. types of content accessed).

Implementation will further be assessed through implementer records of activities and weekly Open Data Kit survey with implementing partner facilitators. Facilitators will record basic participant engagement statistics, as well as brief notes on challenges and successes encountered in programme delivery. To assess the quality of support, a random sample of the live chat sessions in the moderated WhatsApp groups will be monitored. It will be explained to caregivers that the purpose of the monitoring is to assess facilitator skills and ensure that they are getting the best possible service.

#### Intervention acceptability, benefits, and challenges

To complement the quantitative analyses, we will conduct qualitative interviews with adolescents, caregivers, and programme staff. Interviews with caregivers and adolescents will explore participant satisfaction and engagement with ParentApp; participant perspectives on ParentApp’s acceptability and cultural relevance; experiences of delivery, and their perceived benefits and challenges of the programme. Interviews with programme staff will explore their experiences of supporting ParentApp users, benefits, and challenges of delivering the programme. Questions will address delivery contexts and implementation processes, barriers and facilitators experienced during implementation, and recommendations for future implementation. Where appropriate, focus groups will be conducted with staff.

#### Exploratory interviews with siblings

If additional funding is obtained, an additional exploratory set of interviews will take place. Between 1-month post-test and 12-month follow-up stages, adolescent siblings, or other adolescent co-residents in the household to the participating adolescents will be interviewed using the same questionnaire as the 1-month post-test. This would allow exploratory endline-only analysis to begin to understand whether a parenting intervention could have benefits for more than one young person in a household.

### Participant timeline {13}

**Fig. 1:**
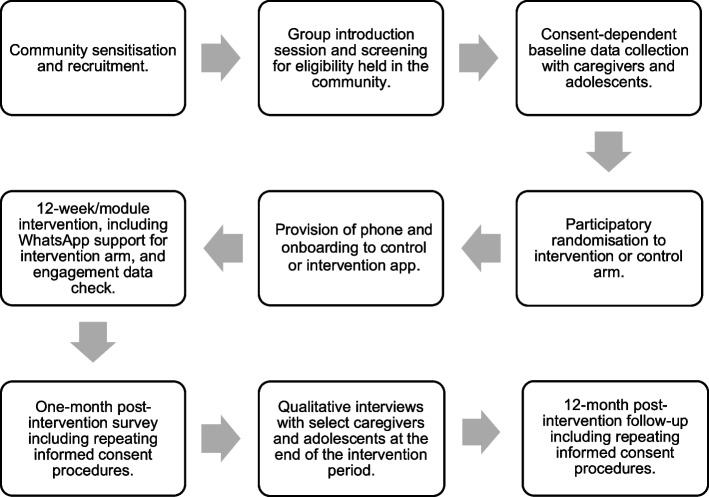
Participant flow chart

**Fig. 2:**
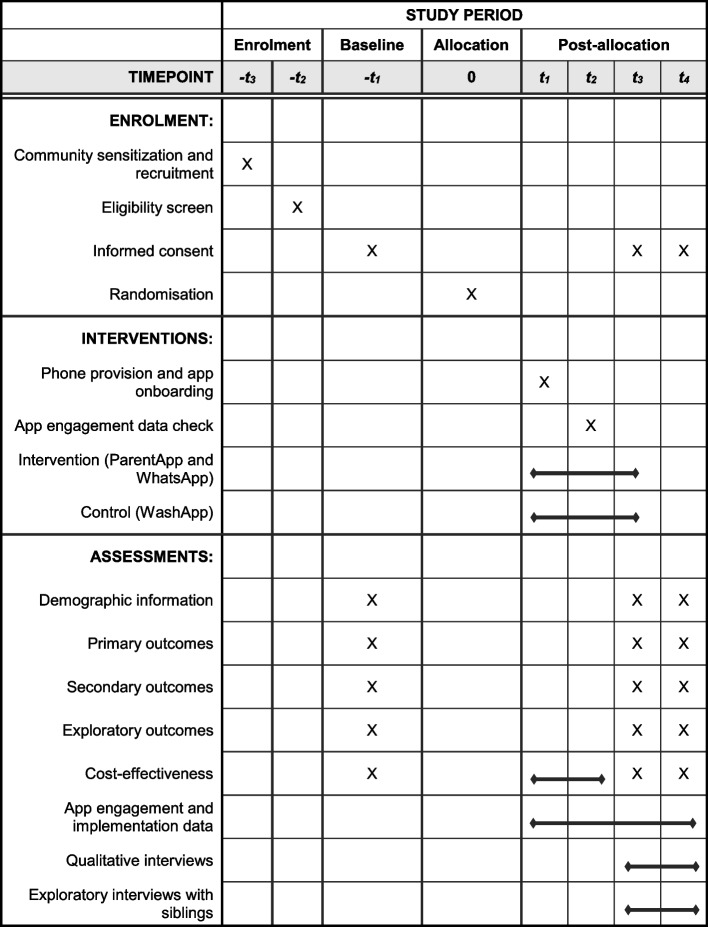
Schedule of enrolment, interventions, and assessments

The participant flow chart can be seen in Fig. 1 and the overall participant timeline in Fig. 2. There will be a rolling recruitment and enrolment strategy, with recruitment efforts ending when the full sample of caregiver-adolescent dyads have been successfully identified and enrolled in the study; we anticipate a timeline of 4–6 months to reach the intended sample size. The first post-intervention data collection will take place around week 16, following participant engagement in the intervention. Further follow-up surveys will be conducted approximately 16 months after beginning the apps. At follow-up, participants will be asked for consent to contact them again in the future, in case of longer-term follow-up.

### Sample size {14}

The study will recruit approximately 2400 caregiver-adolescent dyads (i.e. 2400 caregivers and 2400 adolescents) to take part in the RCT; of these, between 40 and 60 caregivers and between 20 and 30 adolescents will also participate in an individual post-programme qualitative interview. The sample size was determined by power calculations from the pilot study, using multilevel Poisson regression.

modelling. These suggest that if the outcome scale generates a control group mean of 4 and an effect size of 0.95, and accounting for attrition in both the trial and the programme, to get 80% power a minimum of 80 clusters with 30 families in each cluster is required (i.e. 2400 caregiver-adolescent dyads). Semi-structured qualitative interviews will also be conducted with 10 staff responsible for programme delivery at each of the implementing organisations. Adolescent siblings or co-residents in the household in an estimated 50% of households will be assessed at endline only.

### Recruitment {15}

Recruitment follows pragmatic trial principles of closely approximating government scale-up. We will work via established partnerships with UNICEF and the Tanzanian government, through NIMR, and local implementing partner organisation, ICS. Recruitment of caregiver-adolescent dyads will involve a range of strategies, including recruitment through existing groups (such as farmer’s groups), dissemination of posters and flyers in the community, word-of-mouth referral and/or online recruitment methods. Approval will first be sought from local community and political leaders before recruitment activities commence, and they will be closely involved in community mapping and recruitment efforts in their respective communities to identify caregivers who meet the recruitment criteria.

## Assignment of interventions: allocation

### Sequence generation {16a}

The study will use a stratified cluster randomisation procedure. Cluster randomisation will be stratified by cluster location (urban and peri-urban) and use a block size of six clusters. The stratum will be used in the analysis and included as a pre-planned covariate, the percentage of males in the cluster.

### Concealment mechanism {16b}

The Trial Statistician will generate 100 possible allocations for each block, where each block is composed of sites from the same stratum (i.e. urban, or peri-urban). One allocation from the possible allocations within a given block will be chosen at random during a community randomisation meeting using a participatory approach. The identities of the sub wards/clusters will be concealed to the statistician calculating the randomisation.

### Implementation {16c}

The fieldwork team will collect baseline data in blocks of six clusters that are all either (a) urban or (b) peri-urban. Once baseline data has been collected for a block of six clusters, the fieldwork team will hold a collective randomisation ceremony to allocate the sites in that block (i.e. randomisation is done as baseline data collection is completed for the four sites in a block). This will be conducted by asking a community participant or leader to randomly select a number, e.g. by picking a slip of numbered paper from a hat.

## Assignment of interventions: blinding

### Who will be blinded {17a}

The implementing partner will notify the participating families of their allocation status after baseline data collection in their cluster is complete to assure that participants are blind to allocation during the baseline assessments. The allocation status of participating families in other clusters will be concealed from participants, thus reducing the potential for contamination. Based on prior trials of parenting programmes, we do not anticipate that blinding will be possible for research staff after baseline data collection as families usually mention their participation in the intervention or control programme. Similarly, of course, participants cannot be blind to their own treatment condition. However, the data analysts will be blinded from condition assignment during analysis.

### Procedure for unblinding if needed {17b}

Not applicable. Participants and research staff will not be blinded after baseline.

## Data collection and management

### Plans for assessment and collection of outcomes {18a}

Quantitative and qualitative methods will be used for data collection.

#### Quantitative data collection

##### Baseline and follow-up surveys

All outcomes of intervention effectiveness will be measured at baseline, 1-month post-intervention, and at 12-month follow-up. Interviews with adolescent siblings or co-resident adolescents will take place at endline only (between 1-month and 12-month follow-up) in households where there is more than one adolescent.

##### App engagement data

Engagement data is collected automatically through the app which is managed by the software developers IDEMS International.

##### Cost-effectiveness monitoring and evaluation survey

Cost data will be collected via the completion of self-administered weekly ODK-based surveys by implementing partner facilitators. Facilitators will record time and data usage of prescribed intervention efforts, as well as basic participant engagement statistics.

#### Qualitative data collection

Qualitative data collection through interviews and focus groups will be undertaken by research team members and trained research assistants. Interviews will be conducted face-to-face in Kiswahili. With participant consent, all interviews and focus groups will be audio recorded in addition to researchers taking back up notes. Data collection, analysis, and reporting will follow consolidated criteria for reporting qualitative research (COREQ) guidelines [68].

### Plans to promote participant retention and complete follow-up {18b}

Participants who complete the survey assessments will receive a small token of appreciation worth up to USD2 for each round of quantitative and qualitative assessment, excluding the baseline. As research activities will take place in participants’ communities or homes, we expect there to be no need for transport for participants (i.e. venues are within walking distance); however, should this change, participants will be compensated for their travel expenses. Participants will be provided with a certificate of participation regardless of whether they are in the intervention or control group and whether they complete the intervention.

As participants are provided with a low-cost smartphone, the research team and facilitators conduct onboarding in-person to handover of the phone, register SIM cards, and orientate participants to the use of the phone. In this session, participants are taken through the basic use of the phone (e.g. how to switch the phone on and off), how to manage data and storage, and internet and phone safety (e.g. passcode protection). The first module of ParentApp or WASH App is used as an exemplar of how to navigate through the phone and app.

The research team and facilitators will meet with participants approximately 6 weeks after the start of the intervention to confirm accurate syncing of participants’ app engagement data. Learnings from the pilot study and cluster-randomised factorial trial indicated that technical issues (e.g. settings on participants’ phones preventing data syncing using mobile data) can result in incomplete engagement data. Identifying and addressing syncing issues early will allow accurate tracking of engagement across the intervention and facilitate participant tracing.

### Data management {19}

The study activities will ensure that research data is stored, backed-up, managed and curated in a secure manner in line with the General Data Protection Regulation (GDPR) and national data protection legislation, as well as local institution data protection policies. Training by the research team will involve data confidentiality procedures, which will include transportation of paper data in sealed envelopes or locked boxes and storage of paper data in locked cabinets. Questionnaires and consent will be administered using password-protected tablets at the field site and data will be automatically uploaded into a secure server through the open-source software platform ODK. Data will be de-identified prior to data analysis to ensure confidentiality of participants with identifiers kept separately from the research data and linked through unique codes. Electronic records from data collection will be exported from the respective platforms to secure servers at NIMR, University of Cape Town, and the University of Oxford. This includes electronic records of surveys, audio recordings, transcripts, and participant tracking lists. Non-electronic data will be stored in locked filing cabinets at NIMR. App engagement data will be uploaded regularly through end-to-end encryption to an IDEMS International server. Data will then be de-identified and shared with dedicated members of the research team at NIMR, University of Cape Town, and University of Oxford by being uploaded to secure servers at the Universities of Oxford and Cape Town.

### Confidentiality {27}

We will use a unique participant number on research data and app engagement data instead of participant names. Personal identifiers will only be collected for informed consent, operational and logistical purposes (e.g. participant tracing by intervention staff or during follow-up visits). Personally identifiable information (e.g. names, addresses, phone numbers, and contact details of participants) will not be shared with other organisations outside of the research team and will be destroyed after the study unless it is required for follow-up and or future studies and participants have consented to this. Confidentiality will be maintained by delinking all personal identification in the final datasets used for analysis. All anonymised data will be kept for 5 years, in accordance with ethical standards and all non-anonymised data will be disposed of after study completion. Since only de-identified quantitative app engagement data will be provided to the research team, we foresee minimal risks to participants.

### Plans for collection, laboratory evaluation and storage of biological specimens for genetic or molecular analysis in this trial/future use {33}

Not applicable. This study will not collect, evaluate or store biological specimens.

## Statistical methods

### Statistical methods for primary and secondary outcomes {20a}

Quantitative data will be cleaned and analysed in R, R studio and Stata. The primary method of analysis will be via a three-level model with adjustment for person-level characteristics and multiple imputation. In this three-level model, measurement waves are nested within a person, and this is nested within a village or residential area (i.e. cluster). Each regression will include at level 3, a term for intervention allocation, the variable used to stratify randomisation, percentage of male caregivers in the village sample, and, as appropriate, the mean of any additional level 2 covariates; at level 2, variables for individual demographic characteristics (e.g. gender, age, disability, caregiver-child relationship) centred at the overall sample mean; and at level 1, a variable for categorical time and interactions between intervention allocation and categorical time (i.e. the test of intervention effectiveness). In situations where more than one caregiver or adolescent is recruited from a family, a clustered standard error structure will be implemented on level 2. The regression link function will be based on consideration of outcome distributions. For example, where outcomes are binary, a logit link will be used; where outcomes take on integer values from 0 and are count-distributed, then a Poisson distribution will be used. We will also produce unadjusted multiply imputed estimates for each outcome, including only terms for intervention, stratification, time, and intervention by time; and unadjusted unimputed estimates for each outcome, following the same specification.

The *p*-value threshold will be 0.05 for all pre-specified analyses. However, due to multiple comparisons planned for exploratory analyses, a sharpened *q*-value will be used with respect to all exploratory analyses to adjust the *p*-value threshold for statistical significance to reduce the likelihood of false positives. False discovery will be controlled using a testing hierarchy.

The significance of the fixed effects in the model will be evaluated using Wald tests, which assess whether the regression coefficients are significantly different from zero. Overall, intervention effectiveness for a given outcome will also be evaluated via a likelihood ratio test comparing models with intervention by time interactions with models without intervention by time interactions. For random effects, the intracluster correlation coefficient (ICC) may also be used to evaluate the proportion of variance in the outcome variable that is due to variation in the higher-level units. Because ICC is also dependent on variable scale where variables are e.g. binary or Poisson distributed, we will also estimate the median incidence rate ratio or odds ratio as appropriate.

Where variables are approximately normally distributed with respect to covariates, mean differences will be used. Where variables are Poisson-distributed, incidence rate ratios (IRRs), which represent the ratio of the incidence rate or frequency of the outcome in one group compared to another group, will be calculated. The advantage of using IRRs is that they allow for the comparison of rates, frequencies or ratios of means across different time points or groups, while controlling for other covariates. Where outcomes are binary or ordinal, odds ratios will be estimated.

### Interim analyses {21b}

Given the strong policy engagement in ParentApp within Tanzania, and requests by the National Government to scale up the programme, we will conduct and share analyses of results at 1 month post-intervention, as well as at 12 months post-intervention.

### Methods for additional analyses {20b}

Analyses will additionally consider mediation and moderation. Mediation analyses will be undertaken where the intervention reflects a significant effect on a candidate mediator post-intervention and on a primary outcome at final follow-up. Mediation analyses will use a two-level model with people within the village and use a 2–1-1 mediation model [69]. This model will include at level 2, intervention allocation, the cluster-level mean of the mediator at post-intervention, and the cluster-level random intercept of the outcome at final follow-up, controlling for the stratification factor, cluster-level mean of the mediator and cluster-level mean of the outcome at baseline; and at level 1, the post-intervention mediator centred within cluster as a regressor for the outcome at final follow-up. The benefit of this model is that by differencing the cluster-level and person-level pathways between mediator and outcome, any contextual mediation effects can be identified.

Moderation analyses will include the same vector of level 2 characteristics as in main outcome models; however, focal moderators will be tested by centring each moderator within context and testing both level 3 interactions (i.e. whether intervention effects are different based on cluster-level compositional characteristics) and level 2 interactions (i.e. whether intervention effects are different based on individual-level characteristics). If data allows, we will also test moderated mediation hypotheses using a multiple-groups multilevel structural equation modelling approach [70], we will revisit mediation models using level 3 variables as stratifiers to test whether paths comprising indirect effects are different over strata. Final analytic plans for mediation and moderation analyses will be submitted to OSF a priori to conducting these analyses.

Qualitative data will be transcribed verbatim, translated into English, and coded using NVivo 12 software; to aid the analysis, the team will utilise a codebook developed inductively and deductively. Matrices and other visual displays will be used to explore thematic areas.

### Methods in analysis to handle protocol non-adherence and any statistical methods to handle missing data {20c}

We will use a two-level ‘wide’ multiple imputation with fully conditional specifications in Mplus, specifying a model with intervention terms and stratifiers at level 2 and relevant baseline person-level predictors at level 1. This requires ignoring possible clustering of standard errors where, e.g. more than one caregiver is recruited in a family, and may thus generate biased estimates of ICCs. However, the impact of this is likely to be negligible as the substantive variance partition of interest is at level 3.

Analyses will be conducted on an intention-to-treat basis (i.e. we will include everyone who dropped out after enrolment). However, if there is significant dropout, we will exploratorily analyse causal effects using compiler average causal effects of components [71].

### Plans to give access to the full protocol, participant-level data and statistical code {31c}

All components of this research, including but not limited to the research protocol, fully anonymised datasets, code, and data analyses will be made available on the Open Science Framework. Project outcomes will be shared through peer-reviewed publications and actively disseminated to government officials, policymakers, NGOs and communities within Tanzania and globally.

## Oversight and monitoring

### Composition of the coordinating centre and trial steering committee {5d}

A Trial Steering Committee (TSC) has been assembled and will meet annually (or more frequently if required) to ensure the study is conducted with rigour and provide advice to the Principal Investigators (PIs), research team and implementing partners. The TSC is composed of independent international and Tanzanian experts in scientific research, violence against children, local community and implementation, as well as a local parent representative. It holds responsibility for oversight of trial progress against the protocol, respondent safety and consideration of new information of relevance to the research objectives.

### Composition of the data monitoring committee, its role and reporting structure {21a}

A data monitoring committee was not established, as this is considered a low risk pragmatic trial of a social intervention. Should the TSC deem it necessary, a data monitoring committee will be convened during the trial.

### Adverse event reporting and harms {22}

This study commits to the universal principles of human research ethics, respect for persons, beneficence and justice. These will be upheld throughout the various stages of the trial, including recruitment, onboarding, data collection and app engagement. There is a possibility that participants may disclose experiences of ongoing violent practices, whether as the recipient or perpetrator of harm towards themselves, their child(ren) and/or partner. Within participant informed consent, it is made clear that some information regarding harmful practices may have to be disclosed if the participant and/or a family member are at risk of harm. The research team, including research assistants and implementation facilitators, will receive training on how to respond to these situations following agreed upon protocol. Finally, if we determine that respondents or their families have experienced significant harm as a result of participation in the research study (i.e. abuse, suicidality, intimate partner violence, or other potential psychological or physical injuries), we will cease further activities until these issues can be addressed adequately.

### Frequency and plans for auditing trial conduct {23}

There is no independent auditing process. A Project Management team and Data Management team, composed of multi-partner research and implementation representatives, each meet weekly and hold joint monthly meetings to monitor trial conduct and data quality. The TSC provides oversight through their annual meetings.

### Plans for communicating important protocol amendments to relevant parties (e.g. trial participants, ethical committees) {25}

Substantial protocol amendments will be communicated to the ethical approval bodies of the University of Oxford, NIMR, and the University of Cape Town, as well as the TSC and relevant online trial registry. Should such amendments concern study participants, these will be communicated through the local implementing partners and additional consent will be sought where necessary. Non-substantial amendments will be kept on record.

### Dissemination plans {31a}

NIMR, the Universities of Cape Town and Oxford, and IDEMS International will utilise study findings to improve the intervention. The study findings will be shared with a wide range of policymakers, government stakeholders, local NGOs, community-based organisations, and community members; findings will also be published in journals and reported at conferences. Our publications approach emphasises providing opportunities for the whole team, including early career researchers, local staff, NGOs and government bodies involved to publish from and distribute findings. In reporting the findings of this study, we will omit names and identifying details, and only report broad locations in which the study took place.

Feedback to participants is a key part of our approach. We will create ‘brief reports’: summaries of study findings in lay language. These will focus on findings relevant to NGOs and government in their work with caregivers of adolescents. Researchers will also report back verbally to participants through community meetings, encouraging their thoughts and feedback on emerging findings. Presentations will be made to local NGOs, health services and community groups. No identifiable details will be given in any dissemination or feedback.

## Discussion

This is the first known randomised trial of a digital parenting programme for families with adolescents in a low- or middle-income country. It follows pragmatic trial principles [47] to test the programme as it would be delivered through government and NGOs in the context of Tanzania. WHO’s INSPIRE ‘Seven Strategies for Ending Violence against Children’ [72] identify that effective parenting programmes are currently the best evidenced approach for prevention of family violence against children. But in-person programmes need to be complemented by other forms of delivery if we are to be able to reach the scale that is needed to prevent a billion cases of child abuse each year. This trial aims to contribute to building knowledge about how we may be able to do this. It takes place within a broader context of the Global Parenting Initiative, a 5-year research-within-implementation collaboration, which includes a series of trials and quasi-experimental studies of hybrid and digital parenting programmes in Malaysia, Philippines, South Africa, Tanzania, Thailand and Uganda.

The evidence base to date shows that digital platforms may be an effective modality, but the exact features of effective interventions are not yet established [73], yet it is noted that effects are enhanced when family is involved, and theoretical frameworks are incorporated [74]. Overviews have noted that inclusion of theoretical concepts underpinning interventions was linked to increased size effects [75], yet it is unclear exactly how theory-driven approaches contribute. Our study will allow for mapping of a variety of theoretical components to shed light on how they may influence efficacy, operationalising coding schemes such as those developed by Michie and Prestwich [76].

This trial aims to examine the detailed impact of a digital approach and provide insight into mechanisms as well as outcomes to inform scalability and generalisability. Digital approaches will never be the whole solution, but they have the potential to be an important part.

## Trial status

Recruitment to the ParentApp trial began in March 2023 and is anticipated to continue until end September 2023. Baseline interviews started in April 2023 and are expected to be completed by end September 2023. The current protocol is version [2] of [11-August-2023].

## Data Availability

Data sharing is not applicable to this article as no datasets were generated or analysed. Some datasets generated by the study will be made available via an open-access repository once fully anonymised and after the main study findings have been published.

## Abbreviations

CABI: Child and Adolescent Behaviour Inventory
CDC: Centres for Disease Control and Prevention
COREQ: Consolidated Criteria for Reporting Qualitative Research
CWBSA: Clowns Without Borders South Africa
GDPR: General Data Protection Regulation
GPI: Global Parenting Initiative
PLH: Parenting for Lifelong Health
ICAST: International Society for the Prevention of Child Abuse and Neglect Screening Tool
ICS: Investing in Children and Strengthening their Societies
IDEMS: Innovations in Development, Education and the Mathematical Sciences
INNODEMS: Innovations in Development, Education and the Mathematical Sciences
IPV: Intimate Partner Violence
LSHTM: London School of Hygiene and Tropical Medicine
MICS: Multiple Indicator Cluster Survey
MSI: Management Systems International
M&E: Monitoring and Evaluation
NGO: Non-governmental organisation
NIMR: National Institute for Medical Research (Tanzania)
ODK: Open Data Kit
PLH: Parenting for Lifelong Health
PHQ: Patient Health Questionnaire
SIM: Subscriber Identity Module
TASAF: Tanzania Social Action Fund
TSC: Trial Steering Committee
UNICEF: United Nations Children’s Fund
VAWI: Violence Against Women Instrument
WASH: Water, Sanitation and Hygiene
WHO: World Health Organization
